# Burden and outcomes of pediatric acute respiratory distress syndrome among children with sepsis: a cohort study

**DOI:** 10.3389/fped.2026.1762030

**Published:** 2026-02-16

**Authors:** Nawras Asiri, Lama Khaled Bahatheq, Naila Shaheen, Yasser M. Kazzaz

**Affiliations:** 1 Department of Pediatrics, Ministry of National Guards Health Affairs, Riyadh, Saudi Arabia; 2 Department of Pediatrics, King Fahad Armed Forces Hospital, Jeddah, Saudi Arabia; 3 Division of Biostatistics, Department of Population Health, King Abdullah International Medical Research Center, Riyadh, Saudi Arabia; 4 King Saud bin Abdulaziz University for Health Sciences, Riyadh, Saudi Arabia; 5 King Abdullah International Medical Research Center, Riyadh, Saudi Arabia

**Keywords:** children, PALICC-2, pediatric acute respiratory distress syndrome (PARDS), phoenix sepsis score, sepsis, septic shock

## Abstract

**Objective:**

To determine the prevalence, clinical characteristics, outcomes, and mortality risk factors of pediatric acute respiratory distress syndrome (PARDS) among children with sepsis, and to compare pulmonary and extrapulmonary PARDS phenotypes.

**Methods:**

This retrospective cohort study analyzed children aged 0–14 years with Phoenix-defined sepsis admitted to a tertiary pediatric intensive care unit between 2015 and 2023. PARDS was defined according to PALICC-2 criteria. Demographics, illness severity, microbiology, organ support requirements, and clinical outcomes were compared between children with and without PARDS and between pulmonary and extrapulmonary phenotypes. Multivariable logistic regression models were used to identify independent predictors of mortality.

**Results:**

Among 279 children with Phoenix-defined sepsis, 161 (57.7%) developed PARDS. Children with PARDS were younger, had higher PELOD-2 and Phoenix severity scores, and required significantly more mechanical ventilation, vasoactive support, and renal replacement therapy compared with those without PARDS. Mortality was substantially higher in the PARDS cohort (36.6% vs. 7.6%). Model-estimated mortality probability increased stepwise with worsening PARDS severity and was highest among children with both septic shock and severe PARDS. Pulmonary PARDS accounted for two-thirds of cases, whereas extrapulmonary PARDS demonstrated a higher inflammatory burden and more bacterial infections. In adjusted analyses, the presence of PARDS, higher PELOD-2 score, and greater cumulative fluid balance were independently associated with mortality.

**Conclusion:**

PARDS is a common and common complication associated with high risk of pediatric sepsis, associated with severe organ dysfunction, increased support requirements, and markedly elevated mortality. These findings underscore the need for multicenter validation to confirm the epidemiology and risk factors of sepsis-associated PARDS and to guide earlier recognition and management approaches for this high-risk population.

## 1 Introduction

Pediatric acute respiratory distress syndrome (ARDS) is a heterogeneous clinical syndrome of acute hypoxemic respiratory failure arising after a known insult, reflecting inflammatory lung injury and impaired gas exchange. Its pathophysiological features include diffuse alveolar damage driven by dysregulated inflammation, endothelial–epithelial injury (1, 2).

The Pediatric Acute Lung Injury Consensus Conference (PALICC) definition was established in 2015 and later updated by PALICC-2 in 2023, which refined pediatric-specific diagnostic criteria (2, 3). According to the PALICC-2 guideline, pediatric ARDS (PARDS) is defined in children under 18 years who develop acute hypoxemia and new chest-imaging opacities within 7 days of a known clinical insult, not fully explained by cardiac failure or fluid overload, and who meet oxygenation thresholds based on respiratory support. Severity is stratified according to oxygenation thresholds using the oxygenation index/oxygen saturation index and PaO₂/FiO₂ ratios across both invasive and noninvasive modes of support (2).

PARDS arises from a spectrum of direct pulmonary (e.g., pneumonia, aspiration) and indirect extrapulmonary (e.g., sepsis, systemic inflammation) causes (4, 5). Pneumonia has been reported as the leading etiology, accounting for 35%–70% of cases, followed by sepsis, contributing approximately 13.5%–43% (6–8).

Despite advances in respiratory support, PARDS remains associated with substantial mortality and morbidity. Reported mortality ranges from approximately 20% to 50% (5, 7–9). A systematic review and meta-analysis showed a pooled mortality of approximately 33.7% (10). Mortality increases stepwise with PARDS severity and with higher oxygenation index (11) Additional mortality risk factors include extrapulmonary PARDS, higher severity of illness, multiorgan failure, fluid overload, immunodeficiency, and higher ventilator pressures (5–10, 12, 13).

The interface between pediatric sepsis and PARDS remains incompletely characterized. The prevalence of PARDS among pediatric patients with sepsis remains poorly defined, with a few studies showing wide variability ranging from 9% to 83%, largely due to inconsistent definitions used for both PARDS and sepsis (14, 15). In addition, the Phoenix pediatric sepsis definition was recently published in 2024 (16). The aim of this study was to characterize PARDS in sepsis by comparing the epidemiology, clinical characteristics, outcomes, and mortality risk factors among children with sepsis with and without PARDS, and between pulmonary and extrapulmonary PARDS phenotypes.

## 2 Methods

### 2.1 Study design and setting

This retrospective cohort study was conducted at King Abdullah Specialized Children's Hospital (KASCH), a large tertiary pediatric referral center in Riyadh, Saudi Arabia. The Pediatric Intensive Care Unit (PICU) is a 28-bed mixed medical–surgical unit that admits approximately 1,300 patients annually and receives referrals from across the region. The unit provides comprehensive advanced organ support, including invasive and noninvasive mechanical ventilation, continuous renal replacement therapy, and plasma exchange. The crude mortality rate in the general PICU during the study period was approximately 3.6%. The study utilized data from the institutional Pediatric Sepsis Registry. The Pediatric Sepsis Registry prospectively captures PICU admissions with suspected or confirmed infection based on predefined screening criteria (abnormal temperature or white blood cell count, cultures obtained, and initiation of antimicrobial agent). The registry captures comprehensive clinical information including demographics, comorbidities, vital signs, laboratory values, microbiological findings, and therapeutic interventions. It also includes the Pediatric Logistic Organ Dysfunction-2 (PELOD-2) and detailed support data, such as use of mechanical ventilation, vasoactive infusions, and renal replacement therapy.

### 2.2 Ethical approval

The study protocol and use of registry data were approved by the Institutional Review Board of King Abdullah International Medical Research Center. The requirement for informed consent was waived because the analysis involved de-identified retrospective data.

### 2.3 Study population

All patients recorded in the Pediatric Sepsis Registry between May 2015 and December 2023 were screened. Children aged 0–14 years who met the Phoenix-defined sepsis criteria were included in the study.

### 2.4 Definitions

PARDS was defined according to the PALICC criteria through independent manual review of individual patient records. Severity (mild–moderate or severe) was assigned based on PALICC definitions after stabilization of respiratory support for ≥4 h. Reviewers systematically documented the variables required for PALICC-2 case definition. These variables included the presence of new chest imaging opacities, the mode of respiratory support (invasive mechanical ventilation, noninvasive ventilation, or high-flow nasal cannula), and oxygenation metrics used for severity stratification, including oxygen saturation index (OSI) and Spo2/Fio2. The specific PALICC-2 defining respiratory variables used for PARDS diagnosis and severity assignment, stratified by PARDS severity, are summarized in Supplementary Table S1. PARDS was further subclassified as pulmonary (P-PARDS) or extrapulmonary (EP-PARDS) based on the primary source of infection.

For the Phoenix pediatric sepsis score calculation, the most abnormal value for each component within the first 24 h of PICU admission was used for score calculation.

Comorbidities were defined based on documented pre-existing diagnoses in the medical record and categorized as chronic respiratory disease (including asthma, bronchopulmonary dysplasia, and cystic fibrosis), gastrointestinal disease, metabolic disorders, neuromuscular disease, seizure disorder or epilepsy, renal disease (including chronic renal failure and dialysis dependence), immunodeficiency (congenital or acquired), neoplastic disease, and chronic hematological disorders.

Cumulative fluid overload percentage was calculated using admission body weight as the reference denominator. Cumulative fluid balance was determined by summing total fluid inputs and subtracting total fluid outputs over the first 72 h of PICU admission. Fluid inputs included all intravenous fluids, blood products, medications, and enteral or parenteral nutrition. Fluid outputs included urine output, gastrointestinal losses, surgical or chest drains, and net ultrafiltration volumes from continuous renal replacement therapy when applicable.

### 2.5 Statistical analysis

Comparative analyses were performed for two major contrasts: children with PARDS vs. those without PARDS, and P-PARDS vs. EP-PARDS. These analyses were restricted to patients meeting Phoenix-defined sepsis criteria. These comparisons evaluated demographic characteristics, comorbidities, infection sources, illness severity measures, respiratory and organ support requirements, and clinical outcomes including mortality, length of stay, and ventilator-free days. Categorical variables were summarized as frequencies and percentages and compared between groups using the chi-square or Fisher's exact test, as appropriate. For categorical variables with more than two levels (e.g., source of infection), reported *p*-values represent a single global comparison of distributions between groups rather than individual category-level comparisons. Continuous variables were reported as median values with their interquartile ranges and compared using the Mann–Whitney U or Kruskal–Wallis test.

Predictors of mortality were estimated using logistic regression models among patients with Phoenix-defined sepsis. The results were reported as crude and adjusted odds ratios (ORs), 95% confidence intervals (CIs), and corresponding *p*-values. Mortality (yes/no) served as the dependent variable. To analyze mortality among all patients with sepsis, independent variables were selected based on clinical discretion and included the presence of PARDS, septic shock, immunodeficiency, PELOD-2 score on day 1, cumulative fluid balance percentages for the first 3 days, age, and positive bacterial culture. A separate logistic regression model was constructed for patients with PARDS and sepsis.

A mortality heat map was then constructed to visualize mortality across sepsis and PARDS severity categories. Phoenix criteria were applied to the entire Pediatric Sepsis Registry cohort to allow comparison across infection without Phoenix-defined sepsis (Phoenix score <2), Phoenix sepsis (score ≥2), and Phoenix septic shock (cardiovascular dysfunction) categories. Predicted mortality values were generated using a multivariable model that included immunodeficiency, PELOD-2 score, cumulative fluid balance percentage, age, and positive bacterial culture as covariates. All analyses were conducted using SAS software, version 9.4, and statistical significance was defined as a two-sided *p*-value < 0.05.

## 3 Results

### 3.1 Prevalence of PARDS in pediatric sepsis

**Figure 1:**
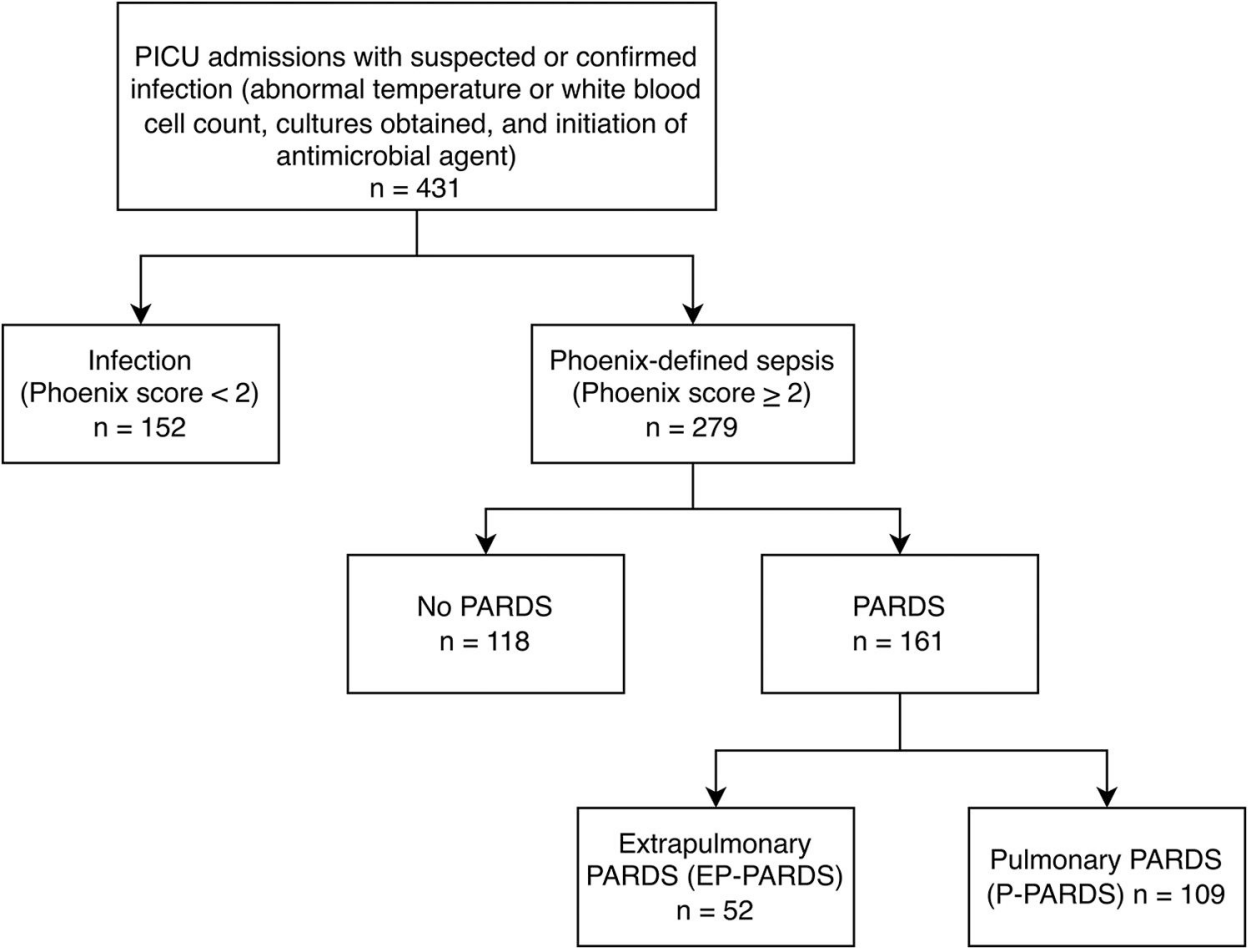
Flow diagram of patient selection and analytic cohorts. The Pediatric Sepsis Registry included 431 PICU admissions with suspected or confirmed infection. Of these, 279 met Phoenix-defined sepsis criteria and comprised the analytic cohort for all comparative and multivariable analyses. PARDS was identified using PALICC-2 criteria and subclassified into pulmonary and extrapulmonary phenotypes. The full registry cohort was additionally used for the mortality heat map analysis to illustrate risk across Phoenix severity categories.

During the study period, 431 PICU admissions were captured in the Pediatric Sepsis Registry. Of these, 279 (64.7%) met Phoenix-defined sepsis and comprised the analytic cohort. Among the Phoenix-defined sepsis cohort, 161 (57.7%) developed PARDS; 109 (67.7%) had pulmonary PARDS and 52 (32.3%) extrapulmonary PARDS (Figure 1). Severity categories among PARDS cohort were distributed as follows: possible PARDS in four patients (2.5%), mild/moderate noninvasive ventilation (NIV) PARDS in 15 patients (9.3%), mild/moderate PARDS in 101 patients (73.2%), severe NIV PARDS in five patients (3.1%), and severe PARDS in 36 patients (26.1%).

### 3.2 Baseline characteristics

Patients with PARDS (*n* = 161) were younger than those without PARDS (*n* = 118) (median age 11.5 months [IQR 3–67.75] vs. [48.5 months (IQR 9–111.25); *p* < 0.001]. Respiratory comorbidity was more common among patients with PARDS (20.3% vs. 9.0%; *p* = 0.003), whereas, immunodeficiency was more common among those without PARDS (11.0% vs. 3.1%; *p* = 0.008). Patients with PARDS were significantly sicker on admission, reflected in both higher PELOD-2 scores [8 (IQR 5.25–11) vs. 5 (IQR, 3–7); *p* < 0.001] and Phoenix severity scores [4 (IQR, 3–6) vs. 2 (IQR, 2–3); *p* < 0.001]. Sex distribution, other chronic comorbidities, and presence of septic shock as per Phoenix score did not differ significantly between groups (Table 1). The laboratory and microbiological characteristics of sepsis patients stratified by PARDS status are presented in Table 2.

**Table 1:** Demographic and clinical characteristics of sepsis patients with PARDS and no PARDS.

Variable	No PARDS (*n* = 118)	PARDS (*n* = 161)	*p*-value
Age (months), median (IQR)	48.5 (9–111.25)	11.5 (3–67.75)	<.001
Sex (M/F)	66 (55.9%)	86 (53.4%)	0.677
PELOD-2 score, median (IQR)	5 (3–7)	8 (5.25–11)	<.001
Phoenix total score, median (IQR)	2 (2–3)	4 (3–6)	<.001
Cumulative Fluid overload percentage	6.9 (2.3–15.5)	14.1 (2.5–23.9)	<.001
Septic shock as per Phoenix, *n* (%)	83 (70.3%)	112 (69.6%)	0.889
Comorbidities, *n* (%)			
Any comorbidity	74 (62.7%)	98 (60.9%)	0.755
Prematurity (<37 wk)	14 (11.9%)	27 (16.8%)	0.253
Immunocompromised	13 (11.0%)	5 (3.1%)	0.008
Asthma	7 (5.9%)	16 (9.9%)	0.229
Other respiratory comorbidities	3 (2.5%)	19 (11.8%)	0.003
Gastrointestinal disease	23 (19.5%)	24 (28%)	0.104
Metabolic disease	9 (7.6%)	12 (7.5%)	0.957
Neoplastic	1 (0.8%)	0 (0%)	0.423
Renal	11 (9.3%)	20 (12.4%)	0.416
Hematologic disorder	15 (12.7%)	11 (6.8%)	0.095
Neuromuscular	7 (5.9%)	10 (6.2%)	0.923
Seizure disorder/Epilepsy	27 (22.9%	33 (20.5)	0.632
Combined comorbidity count			
None	44 (37.3%)	63 (39.1%)	0.470
One	41 (34.7%)	48 (29.8%)	
Two	25 (21.2%)	29 (18.0%)	
Three	7 (5.9%)	19 (11.8%)	
Four	1 (0.8%)	2 (1.2%)	
Source of infection, *n* (%)			
Primary bloodstream infection	9 (7.6%)	0 (0.0%)	<0.001
CNS: meningitis/encephalitis/brain abscess	10 (8.5%)	9 (5.6%)	
Respiratory/Pneumonia	50 (42.4%)	109 (67.7%)	
Abdominal/Gastroenteritis	12 (10.2%)	18 (11.2%)	
Genitourinary infection	6 (5.1%)	3 (1.9%)	
Osteomyelitis/Arthritis	0 (0.0%)	3 (1.9%)	
Skin/Soft tissue infection	5 (4.2%)	9 (5.6%)	
Other	19 (16.1%)	10 (6.2%)	
Head/eyes/ears/nose/throat	7 (5.9%)	0 (0.0%)	

IQR, interquartile range; M/F, male/female; PELOD-2, Pediatric Logistic Organ Dysfunction-2 score; wk, Weeks (gestational age); *n* (%): Number (percentage); CNS, Central Nervous System; PARDS, Pediatric Acute Respiratory Distress Syndrome; NIV, Noninvasive Ventilation.

**Table 2:** Laboratory and microbiology findings of sepsis patients with PARDS and no PARDS.

Variable	No PARDS (*n* = 118)	PARDS (*n* = 161)	*p*-value
Microbiology			
Bacterial pathogen detected (Yes)	40 (33.9%)	80 (49.7%)	0.018
Source of positive culture			
Blood	12 (30.0%)	32 (40.0%)	0.238
Resp	7 (17.5%)	23 (28.7%)	
Urine	11 (27.5%)	10 (12.5%)	
CSF	0 (0.0%)	1 (1.3%)	
Wound	6 (15.0%)	8 (10.0%)	
Other	4 (10.0%)	6 (7.5%)	
Viral pathogen detected (Yes)	46 (39%)	64 (40.3%)	0.831
Fungal pathogen detected (Yes)	1 (0.8%)	7 (4.4%)	0.080
Laboratory Variable			
PCT (ng/mL)	5.02 (3.15–18.95)	10.48 (3.55–56.52)	0.156
WBC (×10^9^/L)	7.74 (4.50–13.60)	11.60 (6.44–20.15)	0.028
Platelet count (×10^9^/L)	247 (183.5–368)	194 (66.5–366.5)	0.312
CRP (mg/L)	40 (11–148)	39 (2–158)	0.821

Resp, Respiratory sample; CSF, Cerebrospinal Fluid; PCT, Procalcitonin; WBC, White Blood Cell; CRP, C-Reactive Protein.

### 3.3 Organ support requirements

Children with PARDS require substantially more organ support than those without PARDS. The PARDS cohort required greater hemodynamic and renal support, with increased use of inotropes (63.4% vs. 44.4%; *p* = 0.002) and continuous renal replacement therapy (10.6% vs. 0.8%; *p* < 0.001) (Table 3).

**Table 3:** Therapies and outcomes of sepsis patients with PARDS and no PARDS.

Variable	No PARDS (*n* = 118)	PARDS (*n* = 161)	*p*-value
Therapies & organ support			
Inotropes used	52 (44.4%)	102 (63.4%)	0.002
CRRT performed	1 (0.8%)	17 (10.6%)	<0.001
Intubation performed	22 (18.6%)	138 (85.7%)	<0.001
Outcomes			
Mortality, *n* (%)	9 (7.6%)	59 (36.6%)	<0.001
Ventilator-free days (median, IQR)	28 (27.5–28)	15 (0–24)	<0.001
PICU length of stay (days)	3 (2–5)	9 (5–18)	<0.001
Hospital length of stay (days)	11 (6.5–23)	19 (9–47)	<0.001
PICU 28-day free days (median, IQR)	25 (22–26)	8 (0–20)	<0.001

CRRT, Continuous Renal Replacement Therapy; IQR, Interquartile Range; PICU, Pediatric Intensive Care Unit.

### 3.4 Clinical outcomes

**Figure 2:**
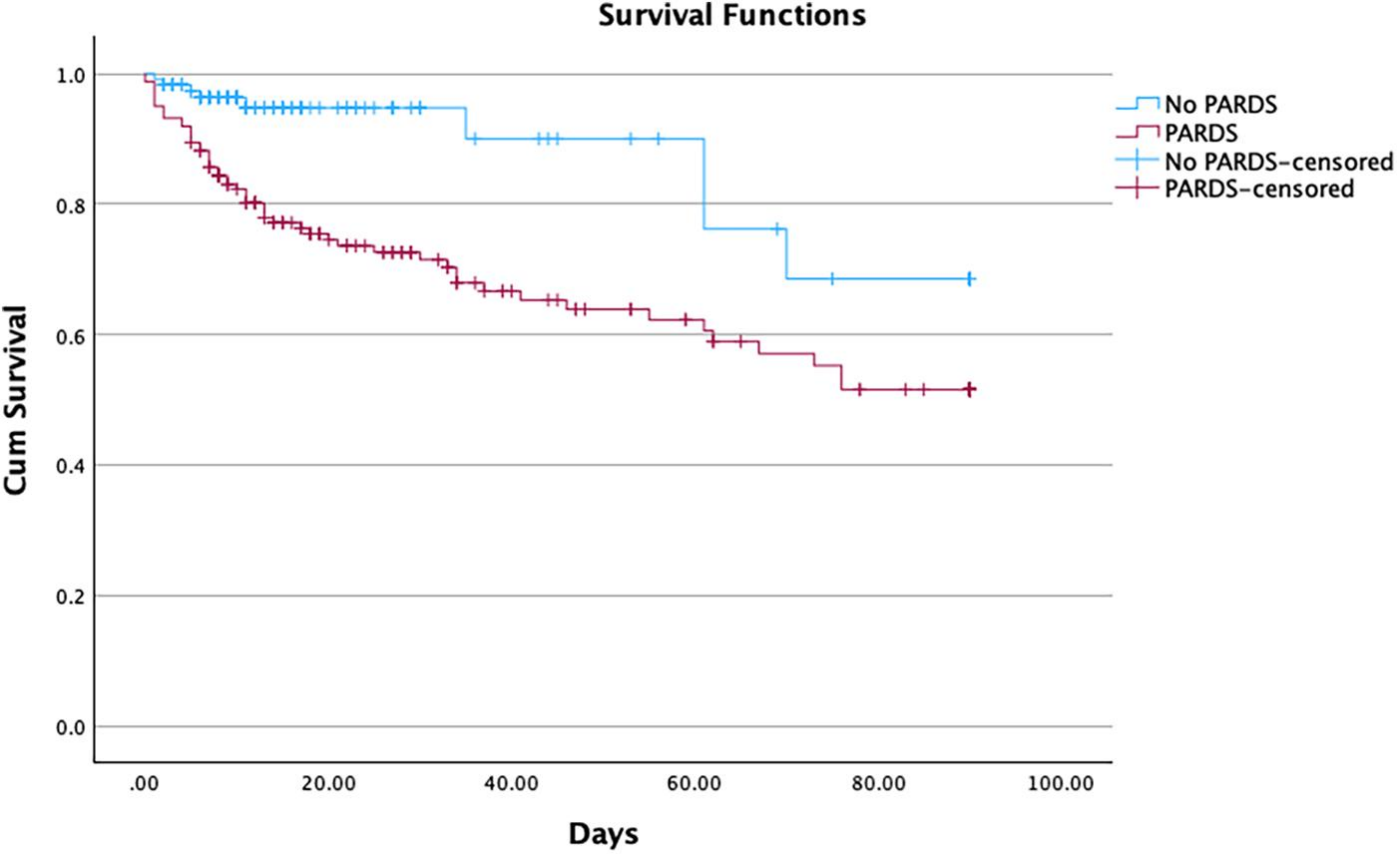
Kaplan–meier survival curves for children with Phoenix-defined sepsis. The Kaplan–Meier survival curves show time-to-mortality among children with Phoenix-defined sepsis, comparing those who developed PARDS with those who did not. Survival was significantly lower in the PARDS group across the follow-up period. Censoring marks indicate patients who were discharged alive.

PARDS was associated with significantly worse clinical outcomes in patients with sepsis. Mortality was significantly higher in the PARDS group (36.6% vs. 7.6%; *p* < 0.001) (Figure 2). Ventilator-free days at 28 days were markedly reduced [15 days (IQR, 0–24) vs. 28 days (27.5–28); *p* < 0.001], as were PICU 28-day free days [8 days (IQR 0–20) vs. 25 days (22–26); *p* < 0.001] in the PARDS cohort. Lengths of stay in both the PICU and hospital were substantially longer in the PARDS cohort (Table 3).

**Figure 3:**
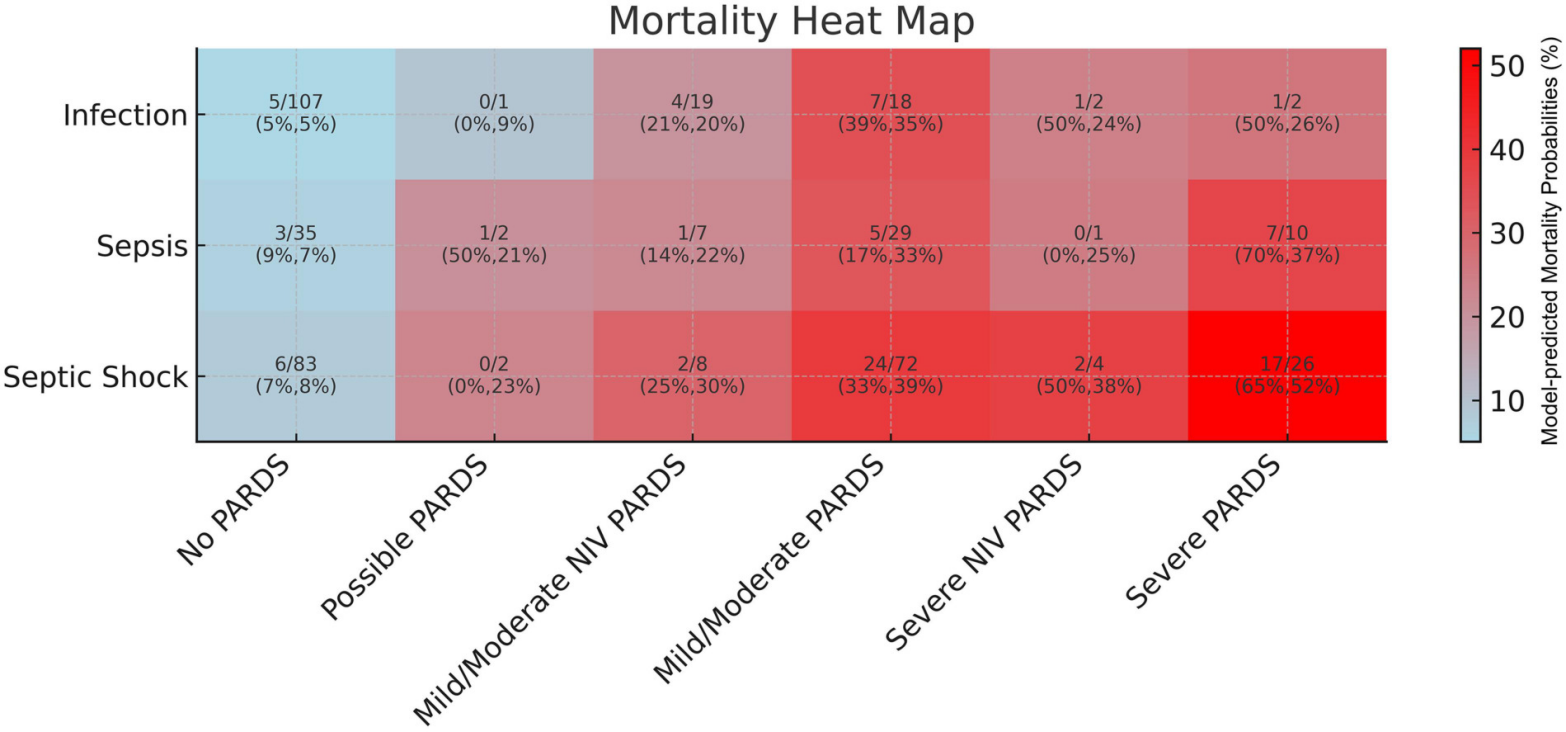
Adjusted estimated probability of mortality by PARDS severity and sepsis severity. The heat map displays the model-predicted mortality probabilities generated from the multivariate logistic regression model (AUC = 0.81). The heat map includes all patients enrolled in the Pediatric Sepsis Registry (*n* = 431), stratified into three Phoenix-based categories: infection (Phoenix score <2), sepsis (score ≥2), and septic shock (cardiovascular dysfunction). The *x*-axis shows the PARDS severity categories and the *y*-axis shows the sepsis severity categories. Mortality probability increased stepwise with worsening PARDS severity. The model covariates included immunodeficiency status, baseline PELOD-2 score, cumulative fluid balance, age, and positive bacterial cultures.

While the primary outcome analyses were restricted to patients meeting Phoenix-defined sepsis criteria (*n* = 279), the mortality heat map additionally illustrates outcomes across the full infection cohort to demonstrate stepwise risk across sepsis and PARDS severity categories. Across PARDS categories, mortality was similar between children with infection and sepsis. In contrast, septic shock was associated with a marked increase in predicted mortality in multivariable regression model, with the highest risk consistently observed in patients with both septic shock and severe PARDS. Across severity levels, NIV-PARDS had mortality rates similar to those of invasive PARDS (Figure 3).

### 3.5 PARDS phenotypes

Of 161 children with PARDS, 109 (67.7%) had P-PARDS and 52 (32.3%) had EP-PARDS. Age, sex distribution, illness severity, and Phoenix sepsis scores were comparable between the two phenotypes (Table 5). Microbiologic profiles differed significantly between phenotypes: bacterial pathogens were detected more frequently in EP-PARDS (63.5% vs. 43.1%; *p* = 0.016), whereas viral pathogens were more common in P-PARDS (48.6% vs. 22.0%; *p* = 0.001). EP-PARDS demonstrated a greater inflammatory burden, characterized by significantly higher procalcitonin and C-reactive protein levels and lower platelet counts (*p* < 0.05) (Table 6). Mortality was higher in EP-PARDS (46.2% vs. 32.1%), though this difference did not reach statistical significance (*p* = 0.084) (Table 7).

**Table 4:** Hazard regression model for PICU mortality.

Predictor	Unadjusted HR (95% CI)	*p*-value	Adjusted HR (95% CI)	*p*-value
PARDS (Yes vs No)	7.01 (3.30–14.85)	<0.001	4.262 (1.871–9.707)	<0.001
Septic shock	0.716 (0.38–1.33)	0.292	0.98 (0.47–2)	0.960
Immunodeficiency	0.88 (0.28–2.77)	0.826	2.567 (0.67–9.85)	0.170
PELOD-2 score	1.18 (1.11–1.26)	<0.001	1.12 (1.04–1.20)	0.003
Cumulative fluid balance %	1.04 (1.02–1.06)	<0.001	1.03 (1.01–1.06)	0.003
Age (months)	0.99 (0.99–1)	0.138	1 (0.99–1.01)	0.918
Positive bacterial culture	1.74 (1.01–2.98)	0.045	1.23 (0.66–2.29)	0.52

CRRT, Continuous Renal Replacement Therapy; IQR, Interquartile Range; PICU, Pediatric Intensive Care Unit; CI, Confidence Interval; *n* (%), number (percentage).

**Table 5:** Demographic and clinical characteristics of patients with P-PARDS and EP-PARDS.

Variable	EP-PARDS (*n* = 52)	P-PARDS (*n* = 109)	*p*-value
Age (months), median (IQR)	7 (2–71.5)	12 (3–66)	0.307
Sex (M/F)	26 (50%)	60 (55%)	0.548
PELOD-2 score, median (IQR)	8.5 (6–12)	8 (5–11)	0.391
Phoenix total score, median (IQR)	4.5 (3–6.75)	4 (3–6)	0.493
Cumulative fluid overload percentage	15.5 (6.3–31.7)	13.7 (6.6–23)	0.324
Septic shock as per Phoenix	33 (63.5%)	79 (72.5%)	0.245
Comorbidities			
Any comorbidity			
Prematurity (<37 wk)	5 (9.6%)	22 (20.2%)	0.093
Immunocompromised	1 (1.9%)	4 (3.6%)	0.480
Asthma	0	16 (14.7%)	0.004
Other respiratory comorbidities	4 (7.7%)	15 (13.8%)	0.264
Gastrointestinal disease	9 (17.3%)	36 (33%)	0.38
Metabolic disease	5 (9.6%)	7 (6.4%)	0.471
Neoplastic	0	0	
Renal	5 (9.6%)	15 (13.8%)	0.456
Hematologic disorder	8 (15.4%)	3 (2.8%)	0.003
Neuromuscular	1 (1.9%)	9 (8.3%)	0.119
Seizure disorder/Epilepsy	6 (11.5%)	27 (24.8%)	0.052
Combined comorbidity count			
None	28 (53.8%)	35 (32.1%)	0.37
One	15 (28.8%)	33 (30.3%)	
Two	4 (7.7%)	25 (22.9%)	
Three	4 (7.7%)	15 (13.8%)	
Four	1 (1.9%)	1 (0.9%)	
Source of infection			
CNS: Meningitis/Encephalitis/Brain Abscess	9 (17.3%)	0 (0.0%)	<0.001
Respiratory/Pneumonia	0 (0.0%)	109 (100.0%)	
Abdominal/Gastroenteritis	18 (34.6%)	0 (0.0%)	
Genitourinary Infection	3 (5.8%)	0 (0.0%)	
Osteomyelitis/Arthritis	3 (5.8%)	0 (0.0%)	
Skin/Soft Tissue Infection	9 (17.3%)	0 (0.0%)	
Other	10 (19.2%)	0 (0.0%)	
PARDS classification			
Possible PARDS	1 (1.9%)	3 (2.8%)	0.384
Mild Moderate NIV PARDS	4 (7.7%)	11 (10.1%)	
Mild Moderate PARDS	32 (61.5%)	69 (63.3%)	
Severe NIV PARDS	0 (0.0%)	5 (4.6%)	
Severe PARDS	15 (28.8%)	21 (19.3%)	

IQR, Interquartile Range; M/F, Male/Female; PELOD-2, Pediatric Logistic Organ Dysfunction-2; CNS, Central Nervous System; PARDS, Pediatric Acute Respiratory Distress Syndrome; P-PARDS; Pulmonary Pediatric Acute Respiratory Distress Syndrome; EP-PARDS, Extrapulmonary Pediatric Acute Respiratory Distress Syndrome; NIV, Noninvasive Ventilation; CI, Confidence Interval.

**Table 6:** Laboratory and microbiology findings of patients with P-PARDS and EP-PARDS.

Variable	EP-PARDS (*n* = 52)	P-PARDS (*n* = 109)	*p*-value
Microbiology			
Bacterial pathogen detected (Yes)	33 (63.5%)	47 (43.1%)	0.016
Source of positive culture			
Blood	21 (63.6%)	11 (23.4%)	<0.001
Resp	0 (0%)	23 (48.9%)	
Urine	2 (6.1%)	8 (17.0%)	
CSF	1 (3.0%)	0 (0%)	
Wound	7 (21.2%)	1 (2.1%)	
Other	2 (6.1%)	4 (8.5%)	
Viral pathogen detected (Yes)	11 (22.0%)	53 (48.6%)	0.001
Fungal pathogen detected (Yes)	2 (3.8%)	5 (4.6%)	0.82
Laboratory Variable			
PCT (ng/mL)	61.25 (13.04–80.41)	4.20 (0.24–40.11)	0.048
WBC (×10^9^/L)	8.52 (4.99–17.38)	13.60 (8.98–21.95)	0.09
Platelet count (×10^9^/L)	62 (44.75–324.50)	197 (120–366.50)	0.001
CRP (mg/L)	215.5 (51.75–267.50)	37.0 (2.00–126.50)	0.01

Resp, Respiratory sample; CSF, Cerebrospinal Fluid; PCT, Procalcitonin; WBC, White Blood Cell; CRP, C-Reactive Protein; P-PARDS, Pulmonary Pediatric Acute Respiratory Distress Syndrome; EP-PARDS, Extrapulmonary Pediatric Acute Respiratory Distress Syndrome.

**Table 7:** Therapies and outcomes of patients with P-PARDS and EP-PARDS.

Variable	EP-PARDS (*n* = 52)	P-PARDS (*n* = 109)	*p*-value
Therapies & organ support			
Inotropes used	37 (71.2%)	65 (59.6%)	0.156
CRRT performed	7 (13.5%)	10 (9.2%)	0.408
Intubation performed	49 (94.2%)	89 (81.7%)	0.033
Outcomes			
Mortality, *n* (%)	24 (46.2%)	35 (32.1%)	0.084
Ventilator-free days (median, IQR)	0 (0–23)	17 (0–24.75)	0.063
PICU length of stay (days)	10 (5.5–19.5)	9 (5–17.75)	0.318
Hospital length of stay (days)	21 (9–66)	19 (9–39.75)	0.482
PICU 28-day free days (median, IQR)	0 (0–17.5)	13 (0–21)	0.056

CRRT, Continuous Renal Replacement Therapy; *n* (%), number (percentage); IQR, Interquartile Range; PICU, Pediatric Intensive Care Unit; P-PARDS, Pulmonary Pediatric Acute Respiratory Distress Syndrome; EPARDS, Extrapulmonary Pediatric Acute Respiratory Distress Syndrome.

The presence of PARDS was independently associated with mortality (adjusted hazard ratio 4.26; 95% CI 1.87–9.71; *p* < 0.001). Higher illness severity and greater cumulative fluid balance percentage were also consistently associated with increased mortality across both the sepsis cohort and the PARDS subgroup (Tables 4, 8).

**Table 8:** Hazard regression model for PICU mortality of PARDS cohort.

Predictor	Unadjusted HR (95% CI)	*p*-value	Adjusted HR (95% CI)	*p*-value
PARDS vs EP-PARDS	0.55 (0.28–1.09)	0.085	0.67 (0.31–1.43)	0.303
Septic shock	1.68 (0.81–3.5)	0.162	1.15 (.5–2.64)	0.74
Immunodeficiency	1.16 (0.19–7.14)	0.874	1.66 (0.25–10.98)	0.6
PELOD-2 score	1.13 (1.05–1.21)	0.001	1.11 (1.02–1.2)	**0**.**012**
Cumulative fluid balance %	1.04 (1.01–1.06)	0.001	1.03 (1.01–1.06)	**0**.**006**
Age (months)	1.00 (0.996–1.008)	0.536	1 (0.99–1.01)	0.361
Positive bacterial culture	1.33 (0.70–2.54)	0.381	1.03 (0.5–2.12)	0.935

PARDS: Pediatric Acute Respiratory Distress Syndrome; P-PARDS: Pulmonary Pediatric Acute Respiratory Distress Syndrome; EPARDS: Extrapulmonary Pediatric Acute Respiratory Distress Syndrome; PELOD-2: Pediatric Logistic Organ Dysfunction-2.

## 4 Discussion

In this retrospective analysis of a prospective cohort of children with sepsis, the prevalence of PARDS was substantial, affecting nearly 40% of Phoenix-defined sepsis cases. PARDS was associated with a five-fold increase in mortality. EP-PARDS demonstrated a predominance of bacterial infections and a markedly higher inflammatory burden; however, these differences did not translate into a statistically significant increase in mortality. Instead, mortality in sepsis-associated PARDS was associated with greater multiorgan failure and higher cumulative fluid balance.

The epidemiology of PARDS in pediatric sepsis remains poorly described, largely because earlier studies did not apply standardized definitions for either syndrome. Estimated prevalence ranges widely from 9% to 83% owing to the heterogeneous criteria used for both conditions (8, 14, 15). A single-center cohort from Indonesia showed a comparable prevalence of 33.9%; however, the study did not specify the sepsis definition applied, and mortality among the sepsis cohort reached 57.1%, exceeding the globally reported rate of 11%–40% (17). In contrast, the present study incorporated structured diagnostic frameworks, enhancing the accuracy and interpretability of PARDS epidemiology within rigorously defined pediatric sepsis and addressing the gap created by the absence of standardized criteria in earlier studies.

In our cohort, PARDS was associated with a five-fold increase in mortality among children with sepsis, with mortality rising in a stepwise manner across PARDS severity categories. This risk is consistent with the findings from multiple cohorts. In a multicenter study across four PICUs in Thailand, children with sepsis-related PARDS had significantly higher mortality than those with nonseptic PARDS (42.3% vs. 17.9%, *p* = 0.016) (8). A higher mortality rate was also reported in a single-center Indonesian cohort, in which 73.7% of children with sepsis-related PARDS died (17). Additionally, a multicenter analysis of the PACCMAN network revealed a mortality rate of 48.8% in EP-PARDS, a subgroup predominantly affected by sepsis (85.4%) (5). Altogether, these studies demonstrate that sepsis-associated PARDS consistently carries a substantially elevated risk of death across diverse healthcare settings.

Mortality in PARDS is driven by an intensified systemic inflammatory response that contributes to both severe respiratory failure and multiorgan injury. PARDS is initiated by neutrophil-mediated alveolar epithelial–endothelial injury, a pulmonary injury (18). This pulmonary injury amplifies the dysregulated immune state of sepsis, worsening cytokine-mediated inflammation, endothelial activation, and generation of reactive oxygen species (4, 5). These mechanisms collectively lead to and worsen multiple organ dysfunction, as demonstrated in our cohort by higher PELOD-2 scores among children with PARDS [median 8 (IQR 5.25–11) vs. 5 (IQR 3–7)]. Similar findings were observed in the PACCMAN multicenter analysis, where extrapulmonary PARDS, predominantly caused by sepsis, was associated with a markedly greater burden of multiorgan failure (5).

In our cohort, EP-PARDS demonstrated a trend toward higher mortality compared with P-PARDS, although this difference did not reach statistical significance. These two phenotypes differed substantially in both etiology and inflammatory burden: EP-PARDS was predominantly associated with bacterial infections, whereas pulmonary PARDS was more frequently viral in origin. Children with EP-PARDS also exhibited higher inflammatory marker levels, consistent with a more intense systemic inflammatory burden and lower platelet counts suggestive of increased platelet consumption. These clinical patterns align with evidence that extrapulmonary lung injury is driven by systemic endothelial injury from circulating inflammatory mediators, whereas P-PARDS reflects primary alveolar epithelial injury, which is more commonly triggered by viral or localized pulmonary pathogens (4).

Furthermore, greater cumulative fluid balance was independently associated with increased mortality in children with PARDS. Similar patterns have been observed in a multicenter study in which children with sepsis-related PARDS had nearly double the degree of fluid overload at 72 h compared with those with nonseptic PARDS [11.5% (5.3–14.6) vs. 6.4% (3.5–9.9)], and this group also exhibited substantially higher mortality (42.3% vs. 17.9%) (8). The detrimental impact of fluid accumulation has also been demonstrated in a large single-center prospective cohort of 290 children with acute lung injury, where each 10 mL/kg/day increase in fluid balance was associated with higher mortality and fewer ventilator-free days. Together, these findings reinforce fluid overload as a key and potentially modifiable determinant of outcomes in sepsis-associated PARDS.

This study has several limitations. First, although sepsis was prospectively identified through a structured registry, PARDS classification relied on retrospective chart review, which may have introduced misclassification despite strict adherence to the PALICC criteria. Second, a key limitation is the absence of detailed ventilator parameters, including tidal volume, PEEP levels, and driving pressure, which prevented assessment of lung-protective ventilation practices. Third, as this was a single-center study conducted at a tertiary referral hospital, our findings may be influenced by local case mix and referral patterns, potentially limiting generalizability. Another important limitation relates to the use of the Phoenix pediatric sepsis criteria. The Phoenix framework was developed primarily to standardize sepsis identification and severity stratification at the population level, rather than as a definitive diagnostic or prognostic tool for individual patients. The aim of this study was not to validate the Phoenix criteria, but to apply this consensus definition to define a sepsis cohort and examine how PALICC-2–defined PARDS interacts with sepsis severity and organ dysfunction phenotypes. Accordingly, our findings should be interpreted as population and phenotype level associations and viewed as hypothesis generating, highlighting the need for future multicenter validation. Finally, residual confounding is possible despite multivariable adjustment, particularly regarding timing of diuresis and renal replacement therapy. Despite these limitations, this study has notable strengths, including the application of the contemporary Phoenix sepsis definition and PALICC-2 PARDS criteria, enabling precise phenotyping that has been lacking in earlier literature. Additionally, the sample size, particularly for a rigorously defined sepsis-associated PARDS cohort is substantially larger than those in previous pediatric studies, allowing for more robust stratified analyses and improved reliability of effect estimates. Collectively, these strengths enhance the validity of our findings and provide a clearer understanding of the epidemiology and outcomes of PARDS in children with sepsis.

In conclusion, PARDS emerged as a prevalent complication linked to substantially increased risk of adverse outcomes. It was associated with greater illness severity, increased organ support requirements, and a five-fold increase in mortality, with risk escalating across PARDS severity categories. EP-PARDS showed features of heightened systemic inflammation, and fluid overload was identified as an important and potentially modifiable contributor to poor outcomes. By applying contemporary sepsis and PALICC-2 PARDS definitions, this study provided a clearer epidemiological profile of sepsis-associated PARDS. Future multicenter studies are needed to validate these findings and to inform early identification and management strategies that may improve outcomes in this high-risk population.

## Data Availability

The raw data supporting the conclusions of this article will be made available by the authors, without undue reservation.
