# Supplementary material for: Burden and outcomes of pediatric acute respiratory distress syndrome among children with sepsis: a cohort study
Source: Front Pediatr. 2026 Feb 16;14:1762030. doi: 10.3389/fped.2026.1762030 (PMC12950753; doi:10.3389/fped.2026.1762030)
Supplement: Supplementary file 1 [file Table1.docx]

Supplementary Table 1. Respiratory Parameters Used for PALICC-2 PARDS Diagnosis and Severity Classification

| **Variable** | **No PARDS (n=118)** | **Mild/Moderate NIV (n=15)** | **Severe NIV (n=5)** | **Mild/Moderate PARDS (n=101)** | **Severe PARDS (n=36)** |
| --- | --- | --- | --- | --- | --- |
| OSI, median (IQR) | 3.6 (3–4.2) | – | – | 7.22 (5.9–9.3) | 20.6 (15.8–28.6) |
| SpO₂/FiO₂, median (IQR) | 285 (280–323) | 227 (200–250) | 135 (100–137) | – | – |
| MAP, median (IQR) | 9 (7–10) | – | – | 13 (11–15) | 22 (16–26.5) |

IQR: Interquartile range; PARDS: Pediatric Acute Respiratory Distress Syndrome; NIV: Noninvasive ventilation; OSI: Oxygen saturation index; MAP: Mean airway pressure.
